# Comparison of VOC Emissions Produced by Different Types of Adhesives Based on Test Chambers

**DOI:** 10.3390/ma14081924

**Published:** 2021-04-12

**Authors:** Mateusz Kozicki, Katarzyna Guzik

**Affiliations:** Department of Thermal Physics, Acoustics and Environment, Building Research Institute, 00-611 Warsaw, Poland; k.guzik@itb.pl

**Keywords:** volatile organic compounds (VOC), construction adhesives, gas chromatography-mass spectrometry TD-GC/MS, indoor air quality (IAQ), temperature

## Abstract

Volatile organic compounds (VOCs) emitted from building materials into the indoor air may cause discomfort associated with a perceptible chemical odour and may irritate the upper respiratory tract. Hence, it is vital to control indoor air pollution sources, such as interior finishing materials, including adhesives. The study involved carrying out a series of experimental tests of VOC emissions of 25 adhesives based on the ISO 16000 series standards. The research concerns three groups of construction adhesives with indoor applications, i.e., flooring (10), finishing walls and ceilings (6), and for other applications such as edge-gluing or gluing tiles or mirrors (9) differing in chemical composition. A series of temperature tests were carried out for a representative floor adhesive at selected temperatures: 25 °C, 35 °C and 45 °C. The theoretical correlation approach was adopted to characterise the relationship between the emission rate and temperature of selected chemical compounds.

## 1. Introduction

According to WHO Housing and health guidelines 2018 [1], indoor air quality is affected by many factors, including the ventilation system, heating, lighting or cooking equipment used, type of furnishing, finishing materials, i.e., adhesives and other coatings, smoking and external contamination. One method of controlling and reducing indoor air pollution sources is to evaluate the products applied indoors concerning their VOC emissions. The products with increased emissions include chemical finishing products such as paints, adhesives, varnishes, and insulation materials.

Concerning adhesives, the WHO report Guidelines for indoor air quality 2010 [2] lists benzene, formaldehyde, tri- and tetrachloroethylene as major emitters. ITB experts’ experience in testing emissions from construction adhesives indicates that construction adhesives emit dozens of different organic compounds, including those potentially harmful to health. The emission values concerning construction adhesives vary and, in some cases, might reach high levels, mainly due to their different base materials.

Another WHO document, The right to healthy indoor air 2000 [3], indicates guidelines for the development of policies and programmes for indoor air for countries where policies and programmes are less developed. This document encourages and promotes the use of international frameworks and principles by governments and other relevant agencies to develop national and local strategies. There is no national system for assessing construction products concerning emissions of volatile organic compounds in Poland, including carcinogenic, mutagenic and toxic for reproduction substances. Nevertheless, some countries use voluntary building rating systems, such as The Blue Angel^®^, EMICODE^®^ or M1^®^. In turn, German AgBB and French VOC Regulations with their emission classes (from A+ to C) are obligatory rating systems [4]. The evaluation procedure for liquid building materials (coatings or adhesives) and decorative products (paint or wallpaper) are proposed by the Protocole AFFSET [5], which contains a list of VOCs with the lower concentrations of interest (LCIs).

According to ECA report No 29 [6], the EU-LCI harmonisation process has led to the development of a protocol for establishing a harmonised list of compounds and EU-LCI values that takes into account existing procedures used in some European countries (e.g., Germany or France). This procedure, based on sound toxicological and risk assessment principles, represents an approach for the evaluation of chemical emissions from construction products. A list of EU-LCI values has been produced [7] whose objective is to establish a common European list of chemicals and their associated toxicological thresholds relevant to human health. The list of EU-LCI values published on this website has been updated annually.

To assess organic compound emissions from construction products, the European Organisation for Technical Approvals (EOTA) Technical Report TR 34 [8] has been developed, which provides guidelines for developing European assessment documents. According to this document, the emission of organic compounds for adhesives and products containing adhesives should be examined.

Apart from the discomfort of staying in a room with a perceptible chemical smell, volatile organic compounds emitted into the air may irritate the upper respiratory tract, cause watery eyes, headaches and malaise. For these reasons, it is important to control potential sources of indoor air pollution, e.g., finishing materials, including adhesives.

Construction adhesives, due to the complexity of their composition and resulting emissions, have an undeniable impact on the presence of chemical compounds that cause indoor air pollution [9,10,11]. What is more, depending on the application, there is a great variability concerning the composition of this group of construction products [12,13]. Broadening the scope of research in the field of construction regarding safety and the environment of use, an attempt was made to systematise and divide adhesives according to their base materials while creating a list of characteristic compounds emitted by adhesives.

The research conducted at the Building Research Institute proved that assembly adhesives are a source of VOC emissions even when applied in very small areas. Thus, for adhesives used indoors, there is a need to test VOC emissions to assess the impact on indoor air quality.

A series of experimental studies on VOC emissions by adhesives with indoor applications have been carried out. The tests were performed for three groups of construction adhesives with indoor applications, i.e., flooring, finishing walls and ceilings, and for other applications such as edge-gluing or gluing tiles or mirrors. The author performed the division of the groups of adhesives based on the Regulation of the Minister Investment and Economic Development of 13 June 2018, amending the Regulation on the performance declaration of construction products and the method of marking them with the construction produce mark [14].

For example, the European standard EN 12004-1:2017 [15] divides cement-based tile adhesives (so only a part of the adhesives described in the paper) into three groups, i.e., cement-based adhesives for fixing tiles on walls and floors inside and outside of buildings, dispersion adhesives and adhesives based on reactive resins for fixing tiles on walls and floors inside of buildings. The standard mentioned above defines classes depending on various characteristics listed in the standard: 1—adhesives with standard bonding; 2—adhesives with enhanced performance, meeting additional requirements concerning adhesion; F—fast setting adhesives; T—slump resistant adhesives; E—adhesives with extended open time; S1—flexible adhesive; S2—highly flexible adhesive.

This thesis aims to systematise groups of adhesives by their emission, application and base materials. The last subdivision is key as it allows for the most precise systematisation of this complex group of construction materials that are adhesives; hence, the following paragraphs briefly describe each of these groups.

Solvent-based adhesives (R) contain flammable organic solvents. A number of them, i.e., benzene, butyl alcohol or cyclohexane, are subject to national regulations concerning emissions [16]. One might see these compounds on the chromatography spectra, and their intensity visibly decreases with the emission time [4]. Once the adhesive is applied, the solvent evaporates relatively quickly, causing an increase in the adhesive viscosity. Bonding can be carried out immediately after applying the adhesive or after the solvent has evaporated, but only before the adhesive has dried and is no longer wet (open time). Waiting for some evaporation to occur before bonding (setting time) increases the adhesive’s initial bonding strength and facilitates faster application.

Polymer-based (P) adhesives are the most diverse group due to the use of various base materials, variations, components and additives to improve the adhesives’ performance. Concerning this group, the authors highlighted a polyurethane (PUR) adhesives subgroup. These adhesives are created as a product of the reaction of different types of isocyanates with polyols. Water reacts with the isocyanate end groups of the prepolymer to form a carbamic acid derivative. It is converted to an amine by eliminating carbon dioxide (CO_2_) and then cross-linked with another isocyanate group to form a polyurea [17]. Because of their extreme flexibility and crack resistance, polyurethane adhesives are also called flexible adhesives.

Polymer hybrid adhesive (PH), otherwise known as a silyl-modified polymer-based adhesive (MS; MSP), is created by combining polymers and silicone. The silyl-modified polymer cures when exposed to moisture, forming a high-performance elastomer. MS polymers have polyether groups in the polymer backbone and contain two active dimethoxy silane groups formed by hydrosilylation of the vinyl terminated middle polymer. The hybrids can contain two or three functional silane groups, which provides additional advantages in terms of mechanical properties [17].

Synthetic rubber-based adhesive (SR) is obtained through the so-called polymerisation reaction. Adhesives contain resins based on thermoplastic, thermosetting polymers and elastomers. There are many different types of synthetic rubbers, including styrene-butadiene rubber (SBR), isobutylene isoprene rubber (IIR or butyl rubber), ethylene-propylene polymers (EPM), polychloroprene rubber (CR), nitrile butadiene rubber (NBR) and silicone rubber [18]. Some rubber-based adhesives require vulcanisation to achieve proper final strength.

Acrylic-based adhesives are suspensions of insoluble resins, which are in the form of fine particles thoroughly mixed with water or organic compounds. The bonding of these types of adhesives is carried out by evaporation of the liquid phase. The addition of catalysts results in better cross-linking of the dispersed particles. There are various types of acrylic-based adhesives, such as cyanoacrylate, UV curing adhesives or waterproof acrylic latex-based adhesives.

Water-based dispersion adhesives consist of a solid component dispersed in an aqueous phase. These adhesives contain water-soluble additives such as surfactants, emulsifiers and protective colloids, which act as bonds between the solid adhesive particles and the aqueous phase. Among this group of adhesives, cellulose adhesives, vinyl cellulose adhesives (mainly used as wallpaper adhesives) and cement-based adhesives (mainly used for bonding ceramic tiles) are distinguished. The latter is composed of cement (e.g., grey or white), mineral fillers, polymers—which provide flexibility—and modifying additives, which determine the setting time. Water dispersion adhesives do not contain organic solvents, thus being safer for human health and the environment.

## 2. Materials and Methods

### 2.1. Emission Testing

**Figure 1:**
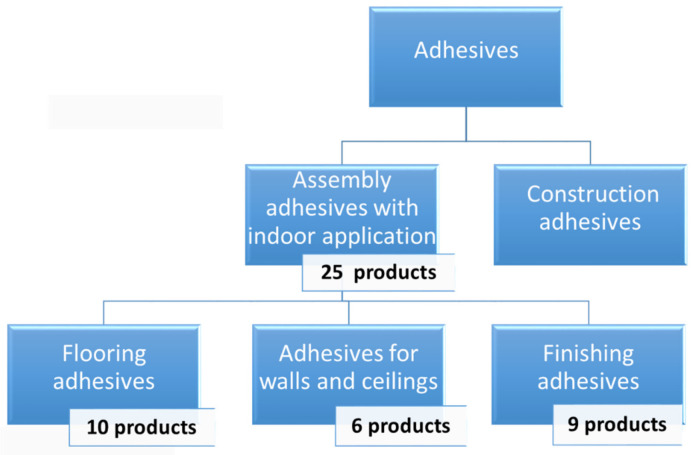
Classification of construction adhesives based on Regulation of the Minister of Investment and Economic Development of 13 June 2018 [14] along with the number of products tested.

Twenty-five different commercially available adhesives were compared by VOC emissions. Tests were carried out on 25 different adhesives, i.e., ten types of flooring adhesives, six types of finishing adhesives for walls and ceilings, and nine types of finishing adhesives, varying in chemical composition (Figure 1). Detailed application of adhesives, declared by manufacturers in tested products datasheets, are presented in Table 1.

The adhesives were tested under the same conditions to ensure reproducible and comparable results. According to the EN 16516: 2017 [19] and EN ISO 16000-9: 2006 [20], one should provide the emissions indicator for day 3 and 28 using the emission test chamber method. The VOC emission testing for these products was conducted in a 0.1 m^3^ test chamber. The test chamber size was appropriate for the intended use of construction products in relation to a reference room described in [19,20]. This room serves as a reference for reflecting actual room conditions.

**Figure 2:**
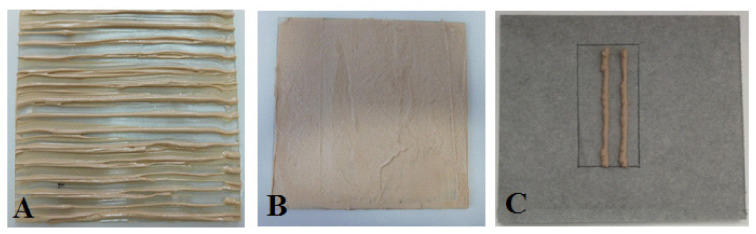
Example of construction adhesive samples prepared for emission testing: (**A**) polyurethane flooring adhesive; (**B**) solvent-based flooring adhesive; (**C**) finishing adhesive.

Each sample was placed on a material inert for emissions (fibre cement panels or glass) (Figure 2) with specific dimensions appropriate to the size of the test chamber under consideration and the product loading factor L (20 cm × 20 cm for floors—L = 0.4; 20 cm × 50 cm for walls—L = 1.0; 10 cm × 5 cm for small areas—L = 0.05). The loading factor is defined as the tested material’s surface area ratio in the reference room to the reference room’s volume (m^2^/m^3^). The relevant product technical sheets shall specify required sampling techniques, taking into account specification and characteristics of the product to be sampled or size of the samples necessary for testing. The thickness and number of the applied adhesive layers shall be consistent with the declared maximum adhesive consumption (product technical data sheets). The manufacturers specified the consumption values in the product datasheets. After the manufacturer’s recommended time (drying time), each sample was placed in an emission chamber. 

The experimental system contained a stainless-steel chamber, a clean air generation and humidification system, an air mixing system, and an environmental monitoring system. The test chamber was connected to an electronic controller, which controlled the test time, airflow and air change rate. To ensure adequate mixing of the air inside the chamber, a small fan was running continuously. The air velocity above the test samples is within the range of 0.1 m/s to 0.3 m/s. The emission chamber was sealed to avoid air exchange with external air. The volume of the chamber was 0.1 m^3^. During the experimental period, the air exchange rate (n) was 0.5 h^−1^, and the temperature inside the chamber was 23 ± 1 °C and 50 ± 5% humidity.

There are many techniques for the isolation and preconcentration of VOCs from air samples, among which the most commonly is a passive method (long-term monitoring) that works by molecular diffusion process and a dynamic method (short-term monitoring) based on a sorption tube [21]. European Standards EN 16,516: 2017 [19] and EN ISO 16,000-9: 2006 [20] indicate reference method for the determination of VOCs and volatile aldehydes emissions from construction products based on test chamber.

VOCs were collected using glass tubes filled with Tenax TA adsorbent, which were desorbed using a thermal desorption unit (TD 20, Shimadzu, Kyoto, Japan). VOCs’ separation and analysis were performed using a gas chromatograph with mass detector (GC/MS-QP2010, Shimadzu, Kyoto, Japan). GC oven temperature programme was used: an initial temperature of 40 °C was maintained for five minutes, followed by a build-up of 10 °C/min to 260 °C, the final temperature was equal to 260 °C, and it was maintained for one minute. An injection mode with a split ratio of 1:10 was used. The method used has a limit of quantification of 1 μg/m^3^. VOCs were identified by comparing the chromatographic peaks’ retention times with those of the reference compounds and matching the resulting compounds’ spectra with those of the NIST 2011 database. The compounds were quantified using the relative response factor obtained from calibration curve standard solutions. Concerning the compounds that were not identified, the total area under the chromatographic curve was converted from molecular weight to concentration using toluene equivalent [22]. Total volatile organic compounds (TVOC) was calculated by summing identified and unidentified compounds eluting between n-hexane and n-hexadecane using the toluene response factor.

### 2.2. Temperature Tests

Temperature tests were carried out at selected temperatures: 25 °C, 35 °C and 45 °C. The duration of each experiment was 77 h. Temperature tests were performed in a 0.225 m^3^ test chamber. The chamber loading was performed according to the EN 16516: 2017 [19] standard and was 0.05 m^2^/m^3^. During the measurement process, samples were taken seven times for each temperature tested. Each time, two samples of five litres of air from the chamber were collected. Temperature tests samples were analysed using TD-GC/MS under identical operating parameters as for the other emission tests.

The uncertainty of the calibrated hytherograph used to measure the temperature in the emission chamber during the experiments. The uncertainty of measurement of the temperature Ut for the range from 20 to 50 °C was 0.2 °C. The expanded uncertainty was calculated using a factor of k = 2 for the level of confidence of approx. 95%.

## 3. Results

### 3.1. Comparison of Adhesives Concerning Their Application 

Laboratory tests results showing TVOC emission levels for individual adhesives and groups of adhesives (flooring adhesives, adhesives for walls and ceilings, and finishing adhesives) after 3 and 28 days after the commencing of the tests are included in Table 2. A summary of all individual compounds emitted along with emission values (μg/m^3^) is presented in the work as Appendix A. 

Table 2 and Appendix A are supplemented by uncertainty for particular compounds (expanded uncertainty k = 2, level of confidence ≅ 95%). Uncertainty was determined based on available data, including data on the accuracy of the measurement system used and experimentally obtained repeatability results. Both tables show average values for two parallel measurements.

It was proved that flooring adhesives (KP) produce the highest emissions among the adhesives with indoor applications tested. It was also the most diverse group of adhesives concerning the emissions. The groups that produced the lowest emission were adhesives for walls and ceilings (KS) (19–72 µg/m^3^).

Among KP adhesives, one could spot that the highest emissions were produced by solvent-based adhesives (675–4184 µg/m^3^) and acrylic-based adhesives (1047–2253 µg/m^3^), while the lowest emissions could be observed for cement-based adhesives (24–40 µg/m^3^).

Finishing adhesives (KW) proved to also be a group with relatively high emissions. In this group, the highest emissions were spotted for solvent-based adhesives (673–1490 µg/m^3^) and hybrid adhesives (126–611 µg/m^3^). The adhesives characterised by the lowest emissivity in this group were acrylic-based adhesives (27–55 µg/m^3^).

### 3.2. Comparison of Adhesives by Base Materials

A division of adhesives based on their base materials has been proposed. The division covers a broad group of adhesives with indoor applications and should be treated only like proposal. This division proposal aims to systematise adhesives in terms of VOC emissions and a chemical basis, which is not available in the literature on the subject. The manufacturers of adhesives do not specify the detailed composition of their products, even the percentage. This is a trade secret, which of course makes it difficult to classify adhesives on the basis of their exact chemical basis.

The area-specific emission rate was determined in order to compare the emission of all tested samples, irrespective of their size. To compare products of different applications and, thus, samples of various sizes tested for VOC emissions, the area-specific emission rate qA (µg/m^2^·h) (also called emission rate) was used. The relationship between qA, the VOC concentration in the exhaust air of the emission test chamber (c) and the airflow rate (q) of the emission test chamber can be expressed as the relationship:

qA = c × q = c × (n/L)(1)

where, L—coefficient of product chamber load factor (m^2^/m^3^), and n—air exchange rate (h^−1^) which was equal to 0.5 h^−1^.

Adhesives for walls and ceilings were tested at L = 1 m^2^/m^3^, flooring adhesives at L = 0.4 m^2^/m^3^, and finishing adhesives at L = 0.05 m^2^/m^3^. A comparison of the qA coefficient for all the adhesives tested shows that the qA depends mainly on the adhesive test’s base material. 

**Figure 3:**
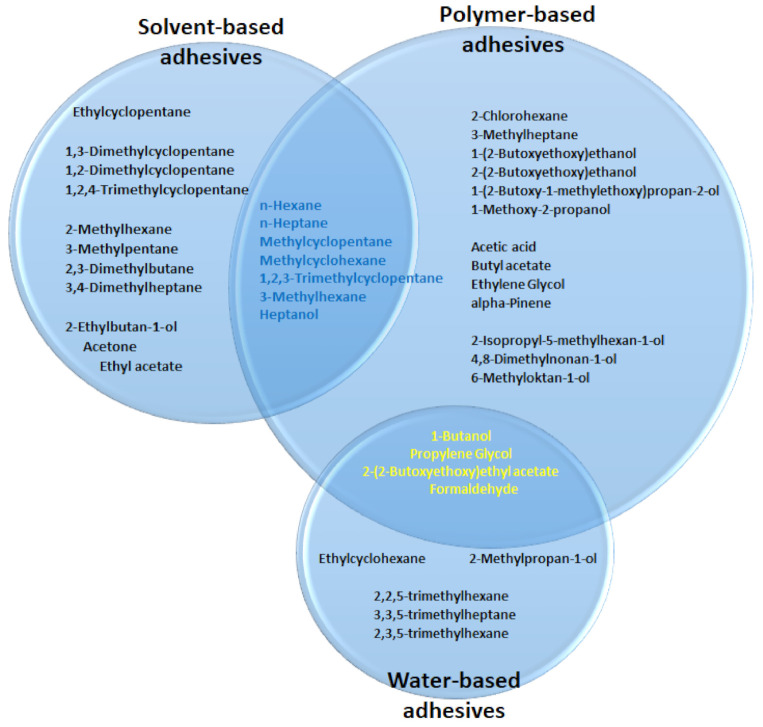
Circle chart of dominant compounds emitted from adhesives made of different base materials. The overlapping fragments contain compounds common for each group.

The tests have shown that adhesives are a diverse group of construction products concerning VOC emissions depending on the base material used. The tested adhesives were divided into three main groups: solvent-based adhesives (R), polymer-based adhesives (P) and water-based dispersion adhesives (DW). The values of the area-specific emission rate qA for all tested groups of adhesives are presented in Table 3 and in the form of a chart—Figure 3.

## 4. Discussion 

### 4.1. VOC Emissions Produced by Adhesives

Analysing VOC emissions data, one might assume that adhesives for wall and ceilings applications are low-emission products. The emission produced by flooring adhesives and finishing adhesives varies depending on the base material used. 

Taking into consideration division by base material, it is clear that the emission concerning the group of solvent-based adhesives (R) was the highest among the groups studied and reached an average value of 8451 μg/m^3^ after three days, while after 28 days, the emission decreased to 2214 μg/m^3^. 

The group of polymer-based adhesives (P) varied the most. Among this group, the highest values could be spotted in the group of acrylic-based adhesives, with an average emission value of 1236 μg/m^3^ after three days and 504 μg/m^3^ after 28 days from commencing the test. The polyurethane adhesives group had the lowest values with mean emissions of 434 μg/m^3^ after three days and 10 μg/m^3^ after 28 days.

Water-based dispersion adhesives (DW) achieved the lowest emission values of all groups tested. Average emission values after three days were 28 μg/m^3^ for methyl cellulose adhesives and 40 μg/m^3^ for cement-based adhesives. After 28 days, the mean emission values for methyl cellulose adhesives decreased to 9 μg/m^3^ and for cement-based adhesives to 13 μg/m^3^.

Analysing the data presented in Appendix A, among the solvent-based adhesives, the most frequently identified groups of chemical compounds were methyl and ethyl derivatives of aliphatic hydrocarbons and cycloalkanes. 

**Figure 4:**
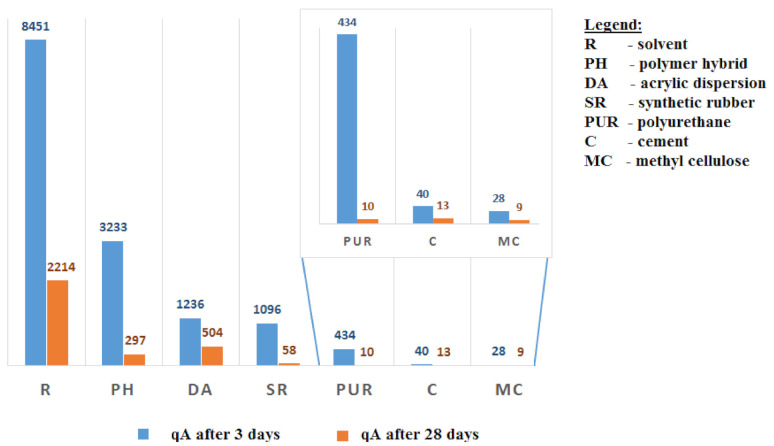
Summary of area-specific emission rate (qA) for all groups of adhesives.

One could see that the greatest variety of emitted compounds is characteristic of polymeric-based adhesives. The most common groups of organic compounds include glycols, their ethers and acetates; simple and branched aliphatic hydrocarbons and alcohols. The least emissive were adhesives based on water-based dispersion, which mainly produced aliphatic hydrocarbons and alcohols (Figure 4).

**Figure 5:**
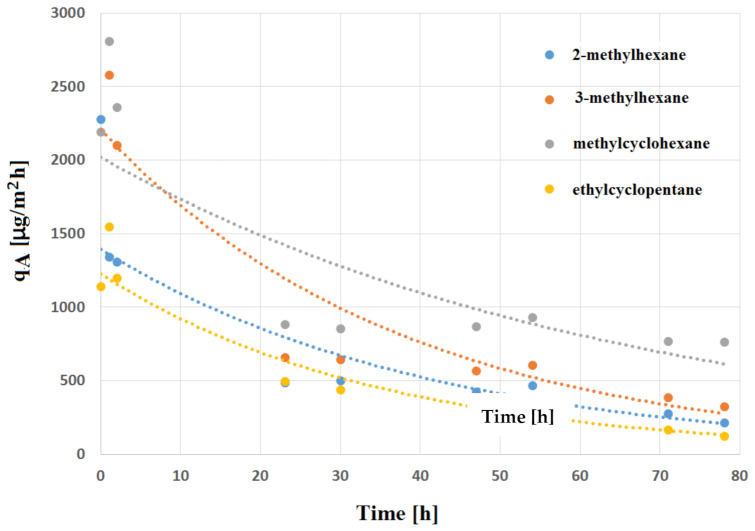
Example of the emission rate of VOC during the 77 h of tests.

The decrease of emission (area-specific emission rate) over time is a typical relationship, observed and widely reported in the literature for different types of construction materials. Emission curves are specific to individual compounds [23,24,25,26]. An example of such a relationship observed after three days for a selected emitted compound, which will be a subject of further analysis, is presented in Figure 5. The highest emission rate in relation to time was observed for 3-methylhexane, while the lowest for ethylcyclopentane. It has been observed that the trend of decreasing concentration over time is similar for all monitored compounds. The highest qA values are at the beginning of the experiment, falling to zero after 77 h of tests.

### 4.2. Temperature Tests

A series of temperature tests at 25 °C, 35 °C and 45 °C concerning flooring adhesive were conducted. 

Numerous empirical and physical models based on concentrations or emission rates in test chambers or indoor air have been developed to characterise VOC emissions from building materials. Most of them are based on three basic parameters, i.e., initial concentration (Co), diffusion coefficient (Dm) and partition coefficient of material and air (K) [27,28,29,30,31].

In this paper, in order to characterise the relationship between the emission rate and the temperature of selected chemical compounds, a theoretic approach to correlation is adopted. This correlation, expressed as the logarithm of the area-specific emission rate intensity by the temperature raised to the power of 0.25, is linearly related to the inverse of the temperature. To show the correlation, the experimental data obtained by carrying out laboratory measurements was used. This study adopts the model developed by Xiong et al. [32], according to which chemical pollutants are emitted from a single-layer building material with uniform diffusivity. What is more, in this model, the material itself must be homogeneous, in that chemical diffusion within the material is one-dimensional and that the air in the emission chamber is well mixed. When the assumed physical process is stabilised, the temperature dependence of the emission can, in a simplified manner, be represented based on dimensionless correlations:

ln (qA/T ^0.25^) = A − B/T (2)

where:

qA—area-specific emission rate [µg/m^2^ h].
T—chamber temperature.
A, B—parameters calculated using experimental data [32] for predicting emission at other temperatures.

This relationship indicates that with increasing temperature, the emission factor increases correspondingly. Therefore, the effect of temperature on the emission rate might be seen as a relationship whereby as the temperature increases, the thermal movement of molecules intensifies, which correspondingly accelerates the diffusion resulting in a higher emission rate. In other words, the concentration gradient between the two sides of the convective boundary layer along the material surface becomes larger, which increases the emission rate [32].

**Figure 6:**
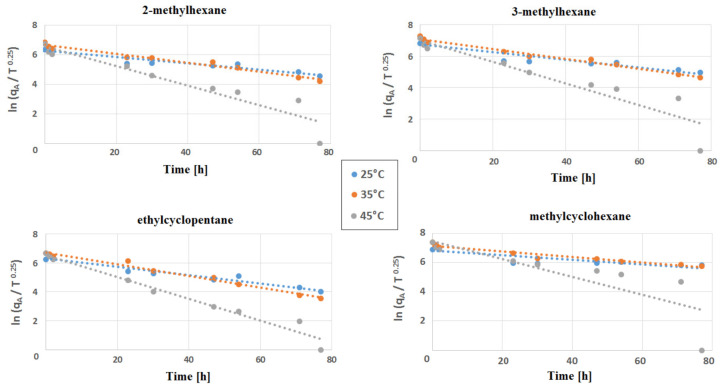
Linear dependence between In (qA/T^0.25^) and time for 2-methylhexane, 3-methylhexane, ethylcyclopenane and methylcyclohexane using obtained data.

A decrease in VOC emissions over time was observed for each of the temperatures tested. Furthermore, from the results obtained, one might conclude that the emissions of the compounds that were monitored (2-methylhexane, 3-methylhexane, ethylcyclopentane and methylcyclohexane) decreased much faster at 45 °C. The linear dependencies for 25 °C and 35 °C are similar and are characterised by a smaller slope (slower decrease). The highest decrease of ln(qA/T^0.25^) value was observed for all investigated compounds at 45 °C. The most dynamic decrease could be observed after 77 h of experiments (Figure 6).

**Figure 7:**
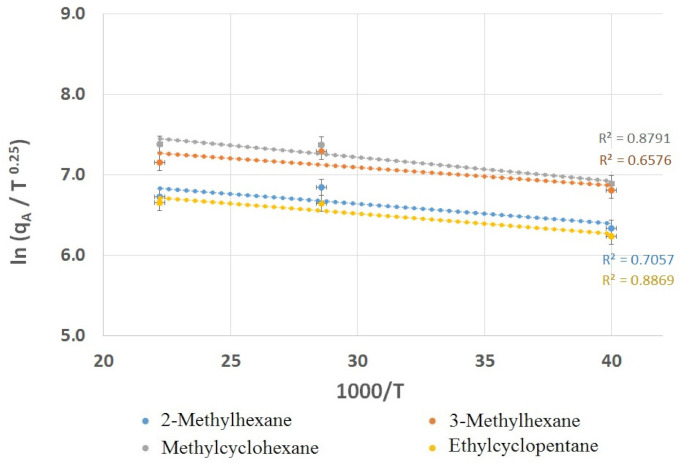
Linear dependence between In (qA/T^0.25^) and temperature for 2-methylhexane, 3-methylhexane, ethylcyclopenane and methylcyclohexane using obtained data.

The data presented in Table 4 shows that emissions at 25 °C reached the lowest values for each compound. The emission values at 35 °C and 45 °C reached similar levels. This is another empirical evidence, illustrated with an example of adhesives, that VOC emission values increase with the increase of temperature. The presented compounds have relatively similar emission rates, ranging from 1141 µg/m^2^·h for ethylcyclopentane to 2193 µg/m^2^·h for methylcyclohexane at 25 °C and from 2020 µg/m^2^·h for ethylcyclopentane to 4155 µg/m^2^·h for methylcyclohexane at 45 °C. However, it is difficult to clearly interpret the results and relate it to the chemical structure of the observed compounds, because the manufacturer does not specify the content of solvents in the tested adhesive. In other words, the In(qA/T^0.25^) values are proportional to temperature, as shown in Figure 7. This relationship is similar to those reported in the literature [33,34,35], which might be considered as evidence of a correctly adopted model and its assumptions. The uncertainty of the hytherograph used to measure the temperature in the emission chamber during the experiments could have influenced the deviation of the correlation coefficients R^2^.

## 5. Conclusions

1. Analysing the data obtained, one might conclude that adhesives for walls and ceilings, regardless of the base material used, are low-emission products. Flooring and finishing adhesives are characterised by high emission values, which vary within their group depending on the base material used.
2. Solvent-based adhesives and acrylic-based adhesives proved to produce the longest lasting emissions (highest concentrations after 28 days of testing).
3. Comparing groups of chemical compounds derived from the tested types of adhesives, the most frequently identified were aliphatic hydrocarbons (including cyclic ones) from the C6–C14 range, glycols, their ethers and acetates, and alcohols.
4. VOC emissions increase with increasing temperature, which means that ln(qA/T^0.25^) values are directly proportional to temperature.
5. The highest decrease of ln(qA/T^0.25^) value was observed for all investigated compounds at 45 °C. The most dynamic decrease could be observed after 77 h of experiments.
6. Characterisation of the material emitting VOCs is important for manufacturers, contractors and building designers to ensure a healthy environment for building occupants. Moreover, manufacturers of adhesives expect information on VOC content to improve product quality, while consumers expect to make informed choices when purchasing them.
7. Verification of finishing materials and products, such as adhesives, is important, as the use of low-emission components will enable erecting low-emission buildings.

## Figures and Tables

**Table 1:** Division of adhesives with indoor applications according to base materials and their application.

Product Type/Symbol		Base Materials	Specific Application (Declared by a Manufacturer)	L (m^2^/m^3^)
Flooring adhesive KP	R 1	synthetic rubber in organic solvents	for parquet gluing	0.4
	R 2	synthetic resin in organic solvents		
	PUR 1	polyurethane adhesives	for floor finishes	
	PUR 2			
	PUR 3		for gluing mosaic parquet, solid parquet, multi-layer parquet, solid planks, industrial mosaic floorings	
	SR 1	hybrid adhesive	wooden floor: parquets (also varnished), solid and glued laminated floorboards, OSB boards, chipboards, plywood, mosaics and wood paving	
	DA 1	acrylic dispersion	for floor coverings, PVC floor covering rolls, tiles and textile coverings	
	DA 2		for gluing textile floor coverings on absorbent surfaces	
	C 1	mixture of cement with mineral fillers and modifiers	for fixing ceramic (glazed tiles, terracotta, gres tiles), cement and natural stone tiles	
	C 2	portland cement	for fixing ceramic (glazed tiles, terracotta, gres tiles, clinker, stoneware, cotto tiles, ceramic mosaic), natural stone and concrete tiles	
Adhesives for finishing walls and ceilings KS	MC 1	methyl cellulose with synthetic resin	vinyl, paper, nonwoven fabric, woodchip or ingrain wallpapers	1.0
	MC 2			
	MC 3		woodchip, vinyl, paper, embossed, silk and textile, acrylic, ingrain wallpapers	
	MC 4	water-based dispersion of methylhydroxyethylcellulose	wall coatings, textile, velvet, and nonwoven wallpapers	
	MC 5	water-based dispersion of methyl cellulose with synthetic resin	textile wallpapers, textile wall coatings, thick wallpapers made of fabric, PVC foil, velvet wallpapers, etc. non-standard applications	
	MC 6	water-based dispersion of methyl cellulose (starch-based)	wallpaper adhesive for gluing paper wallpapers in dry rooms to mineral surfaces	
Finishing adhesives KW	R 3	synthetic rubber in organic solvents	for assembly and finishing works in the construction industry	0.05
	R 4			
	R 5		brick, ceramic, concrete, metal, wood, stone, plasterboard, fibreboard, plywood, MDF	
	PH 1	silyl modified polymer	brick, ceramic, metal, plywood, stone, wood, concrete, plasterboard, fibreboard, MDF, aluminium, mirrors	
	PH 2		wood, brick, ceramic, concrete, plasterboard, stone, metal, glass, mirror, plastic, polystyrene foam	
	PH 3			
	SR 2	styrene-butadiene rubber (SBR)	for gluing mirrors	
	DA 3	acrylic dispersion	wood, polystyrene foam, brick, ceramic, concrete, plasterboard, fibreboard, plywood, stone, MDF	
	DA 4	dispersion resin without lasticisers	for all types of wallpapers	
**Base Materials**				
R—solvent PUR—polyurethane MC—methyl cellulose		DA—acrylic dispersion C—cement	PH—polymer hybrid SR—synthetic rubber	

**Table 2:** TVOC values for adhesives with indoor applications after 3 and 28 days (μg/m^3^).

Product Type/Symbol		TVOC after 3 Days (μg/m^3^)	TVOC after 28 Days (μg/m^3^)	L (m^2^/m^3^)
Flooring adhesive KP	R 1	4184 ± 753	234 ± 42	0.4
	R 2	675 ± 122	199 ± 36	
	PUR 1	202 ± 36	<2	
	PUR 2	80 ± 14	<2	
	PUR 3	760 ± 114	22 ± 3	
	SR 1	482 ± 72	71 ± 11	
	DA 1	2253 ± 406	1211 ± 218	
	DA 2	1047 ± 189	119 ± 21	
	C 1	40 ± 6	21 ± 3	
	C 2	24 ± 4	<2	
Adhesives for walls and ceilings KS	MC 1	59 ± 9	18 ± 3	1.0
	MC 2	51 ± 8	11 ± 2	
	MC 3	72 ± 11	34 ± 5	
	MC 4	65 ± 10	<2	
	MC 5	61 ± 9	44 ± 7	
	MC 6	19 ± 3	<2	
Finishing adhesives KW	R 3	1490 ± 268	428 ± 77	0.05
	R 4	1455 ± 261	459 ± 82	
	R 5	673 ± 101	166 ± 25	
	PH 1	126 ± 19	2 ± 1	
	PH 2	233 ± 35	51 ± 8	
	PH 3	611 ± 92	36 ± 5	
	SR 2	109 ± 16	42 ± 6	
	DA 3	55 ± 8	21 ± 3	
	DA 4	27 ± 4	14 ± 2	
**Application** KP—flooring adhesives KS—adhesives for finishing walls and ceilings KW—finishing adhesives		**Base materials** R—solvent PUR—polyurethane SR—synthetic rubber DA—acrylic dispersion C—cement MC—methyl cellulose PH—polymer hybrid		

**Table 3:** Values of qA for adhesives at 3 and 28 days after commencing the test (μg/m^2^h), together with mean values for each group.

Base Material		Sample Name	q_A_ after 3 Days (µg/m^2^·h)	The Average Value		q_A_ after 28 Days (µg/m^2^·h)	The Average Value		L (m^2^/m^3^)
Solvent-based adhesives (R)		R 1	5230	3037	8451	293	271	2214	0.4
		R 2	844			249			
		R 3	14,900	12,060		4280	3510		0.05
		R 4	14,550			4590			
		R 5	6730			1660			
Polymer-based adhesives (P)	Polyurethane adhesives (PUR)	PUR 1	253	434	434	<1	10	10	0.4
		PUR 2	100			<1			
		PUR 3	950			28			
	MS Polymer Hybrid adhesives (PH)	PH 1	1260	3233	3233	20	297	297	0.05
		PH 2	2330			510			
		PH 3	6110			360			
	Synthetic rubber-based adhesive (SR)	SR 1	603	603	1096	89	89	58	0.4
		SR 2	1589	1589		26	26		0.05
	Acrylic-based adhesive (DA)	DA 1	2816	2062	1236	1514	832	504	0.4
		DA 2	1309			149			
		DA 3	550	410		210	175		0.05
		DA 4	270			140			
Water-based adhesives (DW)	Methyl celluose adhesive (MC)	MC 1	30	28	28	9	9	9	1.0
		MC 2	26			6			
		MC 3	36			17			
		MC 4	33			<1			
		MC 5	31			22			
		MC 6	10			<1			
	Cement adhesives (C)	C 1	50	40	40	26	13	13	0.4
		C 2	30			<1			

**Table 4:** Initial values of emission (C_o_), area-specific emission rate (qA) and ln (qA/T^0.25^) for selected solvents at different temperatures.

Chemical Compound	Temperature (°C)	Concentration Initial C_o_ (µg/m^3^)	q_A_ (µg/m^2^·h)	ln (q_A_/T ^0.25^)
**2-Methylhexane**	25 ± 0.2	1010 ± 242	1263	6.3
	35 ± 0.2	1823 ± 437	2279	6.8
	45 ± 0.2	1735 ± 416	2168	6.7
**3-Methylhexane**	25 ± 0.2	1622 ± 390	2027	6.8
	35 ± 0.2	2852 ± 684	3566	7.3
	45 ± 0.2	2667 ± 640	3333	7.2
**Ethylcyclopentane**	25 ± 0.2	915 ± 220	1141	6.2
	35 ± 0.2	1505 ± 361	1881	6.7
	45 ± 0.2	1616 ± 388	2020	6.7
**Methylcyclohexane**	25 ± 0.2	1755 ± 421	2193	6.9
	35 ± 0.2	3113 ± 747	3891	7.4
	45 ± 0.2	3324 ± 798	4155	7.4

## Data Availability

Data Sharing not applicable.

## Appendix A

**Table A1:** List of main compounds emitted and their emission values (μg/m^3^).

Product Name		Main Compounds Emitted	Emission after 3 Days (μg/m^3^)	Emission after 28 Days (μg/m^3^)
**Finishing** **adhesives** KW Loading Factor L = 0.05 m^2^/m^3^	R 3	2-methylhexane 3-methylhexanen-heptane	844 ± 304	91 ± 16
		1,3-dimethylcyclopentane methylcyclohexane ethylcyclopentane 1,2,4-trimethylcyclopentane 1,2,3-trimethylcyclopentane	429 ± 154	288 ± 51
	R 4	n-hexane 2-methylhexane 3-methylhexane n-heptane	993 ± 179	257 ± 46
		1,3-dimethylcyclopentane methylcyclohexane ethylcyclopentane	432 ± 83	202 ± 36
	R 5	2,3-dimethylbutane	95 ± 14	3 ± 1
		3-methylpentane	41 ± 6	<1
		2-methylhexane	363 ± 54	23 ± 3
		3-methylhexane	374 ± 56	34 ± 5
		2-ethylbutan-1-ol	62 ± 9	<1
	PH 1	2-(2-butoxyethoxy)ethanol	10 ± 2	<1
		2-(2-butoxyethoxy)ethyl acetate	40 ± 6	<1
	PH 2	aliphatic hydrocarbons C6-C14 incl n-hexane	244 ± 37 129 ± 19	<1 <1
	PH 3	aliphatic hydrocarbons C6-C14 incl n-hexane methylcyclopentane	817 ± 123 526 ± 79 11 ± 2	<1 <1 <1
	SR 2	aliphatic hydrocarbons C6-C12 n-hexane methylcyclopentane 3-methylheptane 3-methylhexane 1,2,3-trimethylcyclopentane n-heptane methylcyclohexane butyl acetate	1526 ± 229 116 ± 17 277 ± 41 461 ± 69 282 ± 42 94 ± 14 62 ± 9 115 ± 17 89 ± 13	15 ± 2 <1 <1 <1 <1 <1 <1 15 ± 2 <1
	DA 3	2-hydroxybenzaldehyde	3 ± 1	<1
		1-(2-butoxy-1-methylethoxy)propan-2-ol	3 ± 1	<1
	DA 4	formaldehyde	10 ± 2	<1
**Flooring adhesive** KP Loading Factor L = 0.4 m^2^/m^3^	R 1	2,3-dimethylbutane	239 ± 43	15 ± 3
		3-methylpentane	149 ± 27	14 ± 2
		n-hexane	816 ± 147	11 ± 2
		methylcyclopentane	823 ± 148	66 ± 12
		3-methylhexane	720 ± 130	76 ± 14
		3,4-dimethylheptane	492 ± 89	13 ± 2
		heptanol	133 ± 24	7 ± 1
		1,2-dimethylcyclopentane	81 ± 15	14 ± 3
		n-heptane	446 ± 80	<2
		1-methylcyclohexane	194 ± 35	18 ± 3
		ethylcyclopentane	56 ± 10	<2
	R 2	acetone	458 ± 82	174 ± 31
		n-hexane	132 ± 24	13 ± 2
		ethyl acetate	34 ± 6	< 2
		1-butoxyethoxyethanol	23 ± 4	11 ± 3
	PUR 1	methylcyclopentane	64 ± 10	<1
		2-chlorohexane	63 ± 10	<1
		3-methylhexane	21 ± 3	<1
		heptanol	20 ± 3	<1
		1-methylcyclohexane	15 ± 2	<1
	PUR 2	methylcyclopentane	19 ± 3	<1
		2-chlorohexane	22 ± 4	<1
	PUR 3	1-methoxypropan-2-ol	33 ± 5	<1
		1-(2-butoxyethoxy)ethanol	680 ± 102	<1
	SR 1	propylene glycol	328 ± 49	16 ± 2
		1-butanol	13 ± 2	6 ± 1
		5-methyl-2-(1-methylethyl)hexan-1-ol	21 ± 3	9 ± 1
		4,8-dimethylnonan-1-ol	79 ± 12	46 ± 7
		6-methyloktan-1-ol	27 ± 4	22 ± 3
	DA 1	acetic acid	544 ± 98	63 ± 11
		alpha-pinene	152 ± 27	<1
		dihydro-α-terpineol	130 ± 23	60 ± 11
		2-(2-butoxyethoxy)ethanol	498 ± 90	398 ± 72
		2-(2-butoxyethoxy)ethanol acetate	631 ± 114	596 ± 108
	DA 2	acetic acid	74 ± 14	3 ± 1
		1-butanol	38 ± 7	12 ± 2
		ethylene glycol	825 ± 149	89 ± 16
		alpha-pinene	50 ± 9	6 ± 1
	C 1	1-butanol	18 ± 3	<2
		propylene glycol	22 ± 3	21 ± 3
	C 2	propylene glycol	24 ± 4	<1
**Adhesives for** **finishing walls and ceilings** KS Loading factor L = 1 m^2^/m^3^	MC 1	ethylcyclohexane	14 ± 2	<1
		2,2,5-trimethylhexane	5 ± 1	<1
	MC 2	ethylcyclohexane	14 ± 2	<1
		formaldehyde	9 ± 1	<1
	MC 3	ethylcyclohexane	10 ± 2	<1
		2-(2-butoxyethoxy)ethanol acetate	14 ± 2	3 ± 1
	MC 4	3,3,5-trimethylheptane 2,3,5-trimethylhexane ethylcyclohexane	3 ± 1 2 ± 1 30 ± 5	<1 <1
	MC 5	2-methylpropan-1-ol	16 ± 2	12 ± 2
		propylene glycol	19 ± 3	16 ± 2
		formaldehyde	19 ± 3	<1
	MC 6	unidentified compounds	<5	<5
